# Writing and Reading Histone H3 Lysine 9 Methylation in *Arabidopsis*

**DOI:** 10.3389/fpls.2020.00452

**Published:** 2020-05-06

**Authors:** Linhao Xu, Hua Jiang

**Affiliations:** Leibniz Institute of Plant Genetics and Crop Plant Research, Gatersleben, Germany

**Keywords:** epigenetics, histone, heterochromatin, H3K9 methylation, transcriptional silencing

## Abstract

In eukaryotes, histone H3 lysine 9 methylation (H3K9me) mediates the silencing of invasive and repetitive sequences by preventing the expression of aberrant gene products and the activation of transposition. In *Arabidopsis*, while it is well known that dimethylation of histone H3 at lysine 9 (H3K9me2) is maintained through a feedback loop between H3K9me2 and DNA methylation, the details of the H3K9me2-dependent silencing pathway have not been fully elucidated. Recently, the regulation and the function of H3K9 methylation have been extensively characterized. In this review, we summarize work from the recent studies regarding the regulation of H3K9me2, emphasizing the process of deposition and reading and the biological significance of H3K9me2 in *Arabidopsis*.

## Introduction

In eukaryotic cells, chromatin is divided into two major types of compartments: heterochromatin and euchromatin, reflecting the repressive and permissive potential for transcription in these regions, respectively (Ding et al., 2007). Chromatin is rich in repetitive sequences and transposable elements inside and near centromeres, posing a risk for genome instability through their potential for transposition and meiotic recombination. Thus, during the whole life cycle, it is necessary to keep these regions inaccessible, condensed, and transcriptionally silent. Such regions are classified as constitutive heterochromatin (Saksouk et al., 2015). In contrast, facultative heterochromatin refers to regions whose compaction and silencing are dynamic in the life cycle or under stress stimuli, mainly distributed in chromosomal arms (Trojer and Reinberg, 2007).

Chromatin states are modulated by modifications at the N-terminal tails of histones, DNA methylation, and different histone variants (Jenuwein and Allis, 2001). Histone H3K9 methylation is a critical marker for transcriptional silencing and heterochromatin formation, mostly constitutive heterochromatin formation. Methylation states at H3K9 can be mono- (H3K9me1), di- (H3K9me2), or tri- (H3K9me3) methylation. In mammals, H3K9me3 is the most abundant marker in constitutive heterochromatin (Peters et al., 2003; Rice et al., 2003). However, in plants, the modification of H3K9me1 and H3K9me2 is rich in constitutive heterochromatin and only slightly present in facultative chromatin, whereas H3K9me3 is distributed with a high concentration in euchromatin and at expressed genes (Naumann et al., 2005). In *Arabidopsis*, H3K9me3 methylation broadly marks 40% of all genes (Roudier et al., 2009), but only a low level of H3K9me3 can be detected in regions with transposons and pseudogenes (Charron et al., 2009). Thus, the function of H3K9me3 has been altered in *Arabidopsis* compared to H3K9me3 in yeast and mammals.

H3K9me2 is mainly catalyzed by the histone methyltransferases KRYPTONITE (KYP), SUVH5, and SUVH6 in *Arabidopsis* and is maintained through the feedback loop between H3K9me2 and non-CG methylation (Du et al., 2015). Several studies have shown more details of H3K9me2 deposition with the structural analysis of KYP/SUVH5/SUVH6 and their role in H3K9me2 deposition (Du et al., 2014; Li et al., 2018) and other H3K9 methyltransferases (Caro et al., 2012) and cofactors (Yu et al., 2017). The downstream part of H3K9me2-dependent silencing has also been investigated by identifying a novel H3K9 reader (Zhang C. et al., 2018; Zhao et al., 2019). In this article, we review the writing, reading, and biological roles of H3K9 methylation in *Arabidopsis*.

## H3K9 Methyltransferases in *Arabidopsis*

**FIGURE 1:**
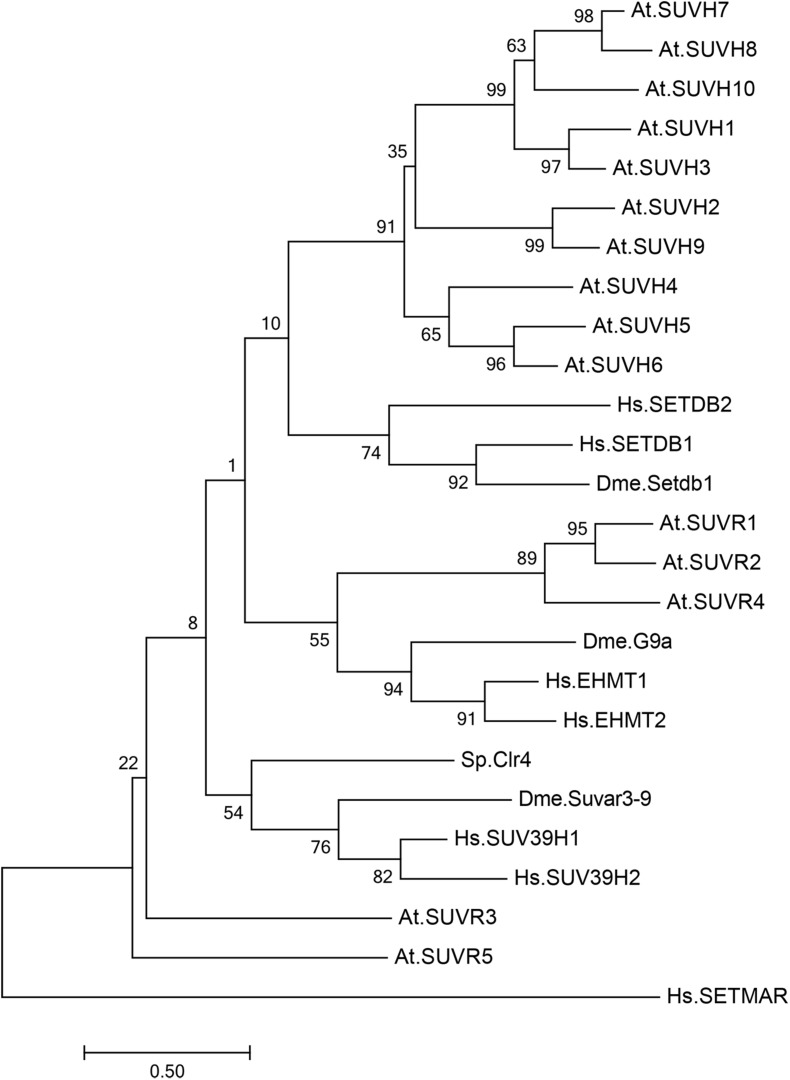
Phylogenetic analysis of SU(VAR)3–9 homologous proteins in *Arabidopsis thaliana*, *Drosophila melanogaster*, *Schizosaccharomyces pombe*, and *Homo sapiens*. Phylogenetic analysis of 15 SU(VAR)3–9 homologous protein sequences from *Arabidopsis thaliana* (At), three SU(VAR)3–9 homologous protein sequences from *Drosophila melanogaster* (Dme), one SU(VAR)3–9 homologous protein sequence from *Schizosaccharomyces pombe* (Sp), and five SU(VAR)3–9 homologous protein sequences from *Homo sapiens* (Hs). The evolutionary history was inferred by using the maximum likelihood method based on the Poisson correction model. Phylogenetic analysis was performed using MEGA 7.0.

Histone lysine methyltransferases usually contain a catalytic SET domain, which is named after three *Drosophila melanogaster* genes, *Su(var)3-9*, *E(z)*, and *Trx* (Jenuwein et al., 1998). In fission yeast, there is only one H3K9 methyltransferase, Clr4/KMT1, which is responsible for all three states of H3K9 methylation (Nakayama et al., 2001; Figure 1). In mammals, there are multiple H3K9 methyltransferases with different catalytic activities and target genes (Sims et al., 2003; Dodge et al., 2004; Shinkai and Tachibana, 2011; Figure 1). SUV39H1 and SUVH39H2 mono- and dimethylase catalyze di-and trimethylation in constitutive heterochromatic regions, SETDB1 monomethylates at the pericentromeric region, and the heterodimer of G9a and G9a-like protein (GLP) catalyzes di- and trimethylation in euchromatic regions.

In *Arabidopsis*, there are 15 SET-domain proteins that are related to SU(VAR)3-9 (Baumbusch et al., 2001; Lei et al., 2012; Zhang and Ma, 2012; Figure 1). Ten of these proteins are classified as SU(VAR)3-9 HOMOLOGS (SUVH1-SUVH9), and the remaining five are classified as SU(VAR)3-9-RELATED proteins (SUVR1-SUVR5) (Table 1). Among the nine SUVHs, KYP/SUVH4, SUVH5, and SUVH6 have been well identified as H3K9 methyltransferases responsible for maintaining H3K9 methylation. KYP mediates the majority of H3K9me2 methylation in both constitutive and facultative heterochromatin in *Arabidopsis*, while SUVH5 and SUVH6 only play minor roles in H3K9me2 methylation (Jackson et al., 2002, 2004; Stroud et al., 2014; Li et al., 2018). Crystal structures of KYP, SUVH5, and SUVH6 reveal that the post-SET domain is critical for enzymatic activity (Li et al., 2018); thus, SUVH2 and SUVH9, which lack the post-SET domain, are enzymatically inactive (Johnson et al., 2014). The remaining SUVH1, SUVH3, SUVH7, and SUVH8 were recently reported to function in transcriptional activation but not silencing, expanding the roles of SUVHs in transcriptional regulation (Harris et al., 2018; Xiao et al., 2019). Nevertheless, SUVH7 and SUVH8 are both primarily expressed and imprinted in the endosperm (Gehring et al., 2011; Wolff et al., 2011), indicating an endosperm-specific targeting mechanism favoring a relatively specific chromatin environment. Indeed, SUVH7 has already been shown to play a role in establishing postzygotic hybridization barriers established by H3K9me2 (Wolff et al., 2015; Jiang et al., 2017). Interestingly, computational characterization predicts that SUVH7 and SUVH8 are capable of catalyzing H3K9me1 and H3K9me2 methylation. Two critical residues in the catalytic pocket, Tyr1124 and Phe1209, determine the product specificity in GLP, a G9a-related methyltransferase (Wu et al., 2010). Meanwhile, H3K9me1 or H3K9me2 is correlated with the presence of Tyr in one of the positions and non-Tyr in the other, indicating that the two SUVHs are capable of catalyzing H3K9me1 or H3K9me2. Thus, SUVH7 and SUVH8 may function as methyltransferases for endosperm-specific H3K9me2 deposition. Taken together, KYP, SUVH5, and SUVH6 are the general H3K9 methyltransferases in *Arabidopsis*, and it is possible that SUVH7 and SUVH8 act as endosperm-specific methyltransferases.

**TABLE 1:** Summary of DNA methyltransferases and SUV methyltransferases in *Arabidopsis thaliana.*

**Gene ID**	**Gene name**	**Description**
AT5G49160	MET1	Maintains CG methylation (Finnegan et al., 1996)
AT4G19020	CMT2	Deposits mainly CHH methylation (Stroud et al., 2014)
AT1G69770	CMT3	Maintains CHG methylation (Lindroth et al., 2001)
AT5G14620	DRM2	Establishes *de novo* CHH methylation (Cao and Jacobsen, 2002)
AT5G04940	SUVH1	Required for transcriptional activation (Harris et al., 2018)
AT2G33290	SUVH2	Recruit RNA polymerase V to establish CHH methylation (Johnson et al., 2008; Johnson et al., 2014)
AT1G73100	SUVH3	Required for transcriptional activation (Harris et al., 2018)
AT5G13960	SUVH4	Maintains H3K9me1/me2 (Jackson et al., 2002)
AT2G35160	SUVH5	Maintains H3K9me1/me2 (Ebbs and Bender, 2006)
AT2G22740	SUVH6	Maintains H3K9me1/me2 (Jackson et al., 2004)
AT1G17770	SUVH7	Paternal-expressed imprinted gene (Gehring et al., 2011; Wolff et al., 2011)
AT2G24740	SUVH8	Maternal-expressed imprinted gene (Gehring et al., 2011; Wolff et al., 2011)
AT4G13460	SUVH9	Recruits RNA polymerase V to establish CHH methylation (Johnson et al., 2008; Johnson et al., 2014)
AT2G05900	SUVH10	Pseudogene (Baumbusch et al., 2001)
AT1G04050	SUVR1	Unknown
AT5G43990	SUVR2	deposits H3K9me1/me2; H4K20me; H3K27me2 (Han et al., 2014)
AT3G03750	SUVR3	Unknown
AT3G04380	SUVR4	Deposits H3K9me2/me3 (Thorstensen et al., 2006; Veiseth et al., 2011)
AT2G23740	SUVR5	Establishes H3K9me2 independently of DNA methylation (Caro et al., 2012)

Among the five SUVRs, SUVR1, and SUVR2 have shown no HMTase activity in an *in vitro* enzymatic assay, but SUVR4 has HMTase activity to convert H3K9me1 to H3K9me2 (ubiquitin) and H3K9me3 (without ubiquitin) *in vitro* (Thorstensen et al., 2006; Veiseth et al., 2011). The level of H3K9me3 is correlated with the amount of SUVR4-GFP in *Arabidopsis* nuclei, but the effect of genome-wide H3K9mer3 has not been determined (Veiseth et al., 2011). SUVR5 is capable of establishing H3K9me2 in a DNA methylation–independent manner and is involved in the response to environmental or developmental cues (Caro et al., 2012).

## Targeting H3K9 Methylation Through Binding to Methylated DNA

**FIGURE 2:**
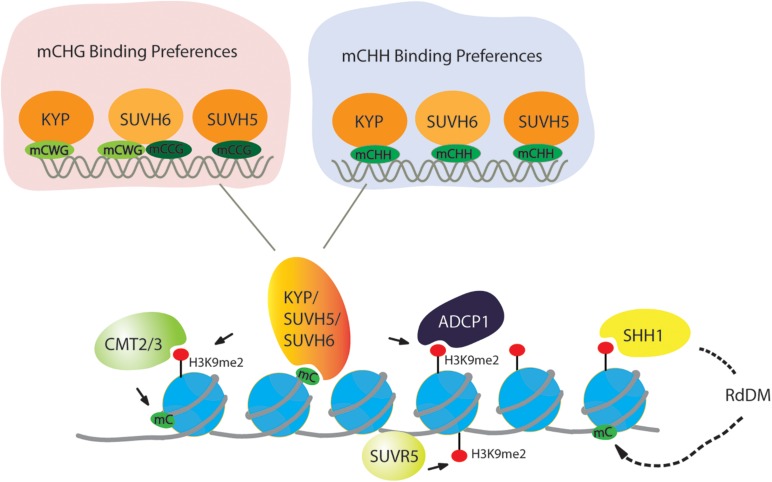
Targeting of H3K9 dimethylation in a DNA methylation-dependent and -independent manner. For DNA methylation, CMT2 and CMT3 recognize the H3K9me2 mark and catalyze DNA methylation in the CHG and CHH context (H = A, T, or C), respectively. For H3K9 methylation, histone methyltransferases KYP/SUVH4, SUVH5, and SUVH6 bind at methylated DNA in the CHG and CHH context to deposit H3K9me2, creating a reinforcing loop between DNA methylation and Histone modification. In the CHG context, KYP has a preference of mCWG (W = A or T), while SUVH5 and SUVH6 have a high affinity to the mCCG. All three SUVHs have similar sequence specificities at mCHH sites. SUVR5 binds to DNA through its zinc finger domain to facilitate H3K9me2 independently of DNA methylation. H3K9me2 is captured by SHH1, then through RdDM pathway to methylate DNA.

DNA methylation is tightly connected with H3K9 methylation. In *Neurospora crassa*, the H3K9 methyltransferase DIM5 establishes H3K9me3, and then heterochromatin protein 1 (HP1) recognizes H3K9me3 to facilitate the targeting of the DNA methyltransferase DIM2 (Tamaru and Selker, 2003). In mammals, knockout of either *G9a* or *Suv39 H1, Suv39 H2* results in reduced DNA methylation in mice (Ikegami et al., 2007). Moreover, H3K9 methylation is dependent on DNA methylation in human cancer cells (Espada et al., 2004). Likewise, in *Arabidopsis*, KYP, SUVH5, and SUVH6 are primarily recruited to the targets through SET and RING-associated (SRA) domain binding to DNA that is methylated in the CHG context (H stands for any base except G). H3K9me2 is known to recruit the DNA methyltransferases CMT2 and CMT3, which mediate CHH and CHG DNA methylation, respectively, in a feedback loop with H3K9me2 (Figure 2; Johnson et al., 2007; Bernatavichute et al., 2008; Du et al., 2012; Zemach et al., 2013; Stroud et al., 2014). Considering the targeting of H3K9 methyltransferases to CHG-methylated DNA, KYP, SUVH5, and SUVH6 have distinct DNA binding preferences. KYP, which is responsible for the majority of H3K9me2, has high affinity to the CWG (W stands for A or T) context but has low affinity to the CCG context (Li et al., 2018). The differential binding affinity is consistent with the phenotype that DNA methylation at CWG is strongly lost in *kyp*, but loss of CCG methylation is very low in *kyp* but high in *suvh5* and *suvh6*. Consistent with the *in vivo* consequence of DNA methylation, SUVH5 has a preference for the CCG context, and SUVH6 can bind to both the CWG and CCG contexts, which act as a backup of KYP to ensure H3K9me2 in all CHG contexts (Figure 2).

While the feedback loop between CHG DNA methylation is frequently discussed, CG and CHH methylation also contribute to H3K9me2 deposition through SRA domains of KYP, SUVH5, and SUVH6 binding to DNA that is methylated in CG or CHH context. In a large-scale comparative epigenome analysis, *MET1* was indeed found to be required for the maintenance of CMT2-dependent asymmetric CHH methylation at loci with H3K9me2 (Zhang Y. et al., 2018). Moreover, SUVH5 and SUVH6 can bind to DNA that is methylated in the CG context *in vitro* (Li et al., 2018), supporting the view that CG methylation also contributes to H3K9me2 deposition. In addition to CG methylation, it has been known for many years that CHH methylation generated by the RNA-directed DNA methylation (RdDM) pathway is also involved in H3K9me2 deposition (Wierzbicki et al., 2008; Zheng et al., 2009; Shin et al., 2013; Liu Z.W. et al., 2014). Recent biochemical evidence indeed supports this hypothesis; all three SUVHs can bind to CHH-methylated DNA (Figure 2), and there is no sequence preference among the three SUVHs in targeting CHH-methylated DNA (Li et al., 2018).

Apart from the specificity of the SRA domain on the sequence context, other factors may also affect KYP, SUVH5, and SUVH6 targeting to methylated DNA. It was reported that SUVH4 and SUVH5 prefer to control transposable elements, but SUVH4 and SUVH6 prefer to target transcribed inverted repeat sources of dsRNA. Thus, in addition to DNA methylation states, chromatin state may also govern SUVH activities (Ebbs and Bender, 2006).

## Targeting H3K9 Methylation Independently of DNA Methylation

Apart from DNA methylation-dependent H3K9me2 deposition, there are known exceptions. G9a is one of the primary enzymes for H3K9me1 and H3K9me2 and usually interacts with another enzyme, GLP, to form a heteromeric complex that appears to be a functional H3K9 methyltransferase *in vivo* (Shinkai and Tachibana, 2011). In murine embryonic stem cells (mESCs), H3K9me2 at the newly integrated proviral LTR is reduced in cells with G9a silencing. Since there is no H3K9me2 or DNA methylation at the newly integrated region, *G9a* is considered to be responsible for *de novo* H3K9me2 (Leung et al., 2011). In addition to mESCs, G9a-dependent H3K9me2 has also been associated with gene repression in multiple human cell lines (Chen et al., 2009; Liu C. et al., 2014; Yoshida et al., 2015; Kramer, 2016; Scheer and Zaph, 2017).

Similar exceptions also exist in *Arabidopsis*. It was reported that SUVR5 is able to establish H3K9me2 independently of DNA methylation (Figure 2; Caro et al., 2012). Unlike KYP/SUVH5/SUVH6, SUVR5 does not have the SRA domain which can bind at methylated DNA but relies on a set of three C2H2 zinc fingers in tandem, which can bind at the sequence context of “TACTAGTA” *in vitro*. This motif also occurs at a minor part of transposable elements (TEs) and surrounds substantial genes losing H3K9me2 in *suvr5*, further supporting the role of zinc fingers in targeting H3K9me2 deposition. While H3K9 methyltransferases in yeast or mammals do not contain zinc fingers, DNA binding proteins recruiting H3K9 methyltransferases contain zinc fingers (Kim and Huang, 2003; Fog et al., 2012; Bian et al., 2015). In mammalian cells, ZNF644 has eight zinc finger motifs and WIZ contains 12 zinc finger motifs that are the binding partners of the G9a-GLP complex (Bian et al., 2015). The N-terminus of ZNF644 interacts with the transcriptional domain (TAD) of G9a, but the C-terminus of WIZ interacts with the TAD of GLP to facilitate the targeting of the G9a-GLP complex at specific genomic loci with the preference of the promoter region (Bian et al., 2015). Thus, it seems that targeting H3K9me2 by the zinc finger domain is a conserved mechanism in plants and mammals, but in plants, the zinc finger domain has been integrated into H3K9 methyltransferase. Interestingly, the combination of zinc fingers and a C-terminal SET domain can be found in all plant species (Caro et al., 2012). Thus, SUVR5 depositing H3K9me2 independently of DNA methylation might be conserved in plants. Another exception in *Arabidopsis* is SUVR4, which can bind to ubiquitin through the N-terminal WIYLD domain to facilitate the conversion from H3K9me1 to H3K9me3 *in vitro*, but it is not clear if the WIYLD domain binds to ubiquitin *in vivo* and if this domain binds to histone or other proteins with ubiquitination (Thorstensen et al., 2006; Veiseth et al., 2011). Recently, it was reported that the CRL4DCAF8 ubiquitin ligase is capable of targeting H3 for polyubiquitination at K79 in mice, which may further promote H3K9me2 deposition (Li et al., 2017), suggesting a similar connection between histone ubiquitination and H3K9me3. Taken together, the deposition of H3K9me2 is not only DNA methylation dependent but can also be independent.

## Other Proteins Participate in H3K9 Methylation Deposition

The distribution of histone acetylation is usually anti-correlated with histone methylation, such as H3K9Ac and H3K9me2 (Zhou et al., 2010), indicating that the removal of H3K9Ac or relevant protein complexes functions in H3K9me2 deposition. Histone deacetylation is processed by histone deacetylases (HDACs), which play important roles in chromatin regulation (Liu X. et al., 2014). In mammals, SUV39H1 can interact with HDAC1 and HDAC2 (Vaute et al., 2002). Moreover, transcriptional repression by SUV39H1 is abolished by treatment with the HDAC inhibitor trichostatin A (TSA), indicating that the function of SUV39H1 is dependent on HDAC activity (Vaute et al., 2002). Likewise, in *Arabidopsis*, one of the HDACs, HDA6, also physically interacts with H3K9 methyltransferases KYP, SUVH5, and SUVH6, regulating a group of transposable elements and repetitive sequences (Yu et al., 2017). The mutant with compromised HDA6 has reduced H3K9me2 levels compared with the wild type, suggesting that H3K9me2 deposited by KYP, SUVH5, and SUVH6 is partly dependent on HDA6, but it is not clear that H3K9me2 deposition in *Arabidopsis* is dependent on the level of H3K9Ac at the targets or depends on the interaction between HDA6 and KYP/SUVH5/SUVH6, or perhaps both mechanisms exist in *Arabidopsis*, which has not been clearly dissected to date. Given the remaining H3K9me2 level in the *hda6* mutant, it will be interesting to know if other HDACs are also involved in H3K9me2 deposition in the future.

Matrix attachment regions (MARs) are important for chromatin organization and gene expression (Tetko et al., 2006; Zhao et al., 2014). MARs are stretches of AT-rich sequences that guide the binding of DNA to the nuclear matrix by recruiting MAR-binding proteins. Proteins with AT-hook motifs bind to MARs and play roles in regulating H3K9me2 levels. In *Neurospora crassa*, CHAP, a protein with AT-hook motifs, was demonstrated to recognize heterochromatic regions through AT-hook motifs and to recruit the H3K9 methyltransferase DIM5 to targets (Honda et al., 2016). In *Arabidopsis*, overexpression of AT-hook motif nuclear localized 22 (AHL22) causes delayed flowering time by increasing H3K9me2 at MAR located in an intron of the FLOWERING LOCUS T (FT) locus (Xiao et al., 2009; Yun et al., 2012). AHL16 regulates the expression of the floral repressor genes FLOWERING LOCUS C (FLC) and FLOWERING WAGENINGEN (FWA) by adjusting the H3K9me2 level (Xu et al., 2013). Overexpressed AHL10 increases genome-wide H3K9me2 levels in the endosperm of triploid seeds (Jiang et al., 2017). Consistent with the distribution of MARs that are mainly in chromosome arms, TEs that are methylated by H3K9me2 *via* AHL10 in the endosperm of triploid seeds are usually euchromatic AT-rich TEs (Jiang et al., 2017). To date, it has not been determined how AHLs regulate H3K9me2 levels. While there is no direct physical interaction between AHLs and H3K9 methyltransferase in *Arabidopsis*, AHLs usually interact with HDAC complexes both *in vitro* and *in vivo*, such as AHL22 interacting with HDA1, HDA6, and HDA9 (Xiao et al., 2009) and AHL16 interacting with FVE and MSI5, which are core components of the HDA6 complex (Gu et al., 2011; Xu et al., 2013). Thus, it is possible that AHLs participate in H3K9me2 deposition through interaction with HDACs, especially HDA6. Nevertheless, other chromatin-relevant proteins also occur in AHL complexes, such as SUVH9 in the AHL10 complex. Apart from interacting with the DDR complex and mediating Pol V recruitment in RdDM (Johnson et al., 2014; Liu Z.W. et al., 2014), SUVH9 also interacts with MORC6 and its two close homologs, MORC1 and MORC2, required for heterochromatin condensation and formation of 3D chromatin architecture at SUPPRESSOR OF DRM1 DRM2 CMT3 (SDC) and Solo-LTR loci (Jing et al., 2016). Recently, the mammalian nuclear matrix protein scaffold attachment factor B (SAFB) was found to participate in stabilizing heterochromatin architecture partially through phase separation, which is a phenomenon in which different biological molecules spontaneously separate into two coexisting liquid phases and result in miscellaneous non-membrane-bound cellular compartments. Depletion of SAFB results in more interchromosomal interactions around pericentromeric heterochromatin and a decrease in genomic compartmentalization, which could result from the decondensation of pericentromeric heterochromatin (Huo et al., 2020). Thus, it is also possible that AHLs and MARs participate in H3K9me2 regulation by affecting heterochromatin architecture and phase separation.

## H3K9 Readers in *Arabidopsis*

H3K9 methylation recruits downstream effectors containing specific reader domains to further mediate gene silencing. In metazoans, heterochromatin protein 1 (HP1) is known to read the trimethylated lysine 9 residue of histone H3 (H3K9me3) (Bannister et al., 2001; Jacobs et al., 2001), which is a hallmark histone modification for transcriptionally silenced heterochromatin in mammals (Zeng et al., 2010). HP1 contains a conserved chromodomain (CD) at the N-terminus and a chromo shadow domain (CSD) at the C-terminus (Li et al., 2002). CD is able to directly bind to H3K9me3 (Jacobs et al., 2001). Based on sequence similarity and early biochemistry analyses, the homolog of HP1 in *Arabidopsis*, LIKE HETEROCHROMATIN PROTEIN 1 (LHP1) was first proposed to be the H3K9me reader that mediates H3K9me2-dependent heterochromatic silencing, as it was shown to bind H3K9me2 *in vitro* (Jackson et al., 2002). Nevertheless, several lines of evidence have indicated that LHP1 is a plant-specific PRC1 H3K27me3 reader subunit. The CHROMO domain of LHP1 specifically binds to H3K27me3 but not H3K9me in *Arabidopsis*, and the genome-wide distribution of LHP1 displays significant overlap with H3K27me3-enriched sites (Turck et al., 2007; Exner et al., 2009; Lu et al., 2011). While SHH1, CMT2, and CMT3 have the capability to bind to histones with H3K9me2 (Law et al., 2013; Stroud et al., 2014; Figure 1), their function is to maintain or initiate non-CG methylation but not the downstream H3K9me2 reader; therefore, the reader of H3K9me2 in plants had not been determined until two recent studies were conducted in *Arabidopsis*. Agenet domain (AGD)-containing p1 (AGDP1), also known as ADCP1, appears to be a plant-specific H3K9 reader and functions as an HP1 equivalent protein (Zhang C. et al., 2018; Zhao et al., 2019). The tandem AGDs of AGDP1 can specifically recognize H3K9me2 and unmethylated K4 on the H3 tail (H3K4me0) through two negatively charged surface pockets. In structural studies, AGD12 adopts a tandem Tudor-like conformation, which resembles the human UHRF1 tandem Tudor and *Arabidopsis* SHH1 SAWADEE domains, both of which function as H3K9me2 readers with similar recognition mechanisms (Arita et al., 2012; Cheng et al., 2013; Law et al., 2013). ADCP1 is responsible for H3K9me2-dependent silencing, and the *in vivo* binding site of ADCP1 largely overlaps with the regions enriched by H3K9me2, further supporting that ADCP1 is indeed an H3K9me2 reader (Zhao et al., 2019).

While ADCP1 has been successfully identified, how ADCP1 mediates H3K9me2-dependent transcriptional silencing still needs to be discovered. Given that ADCP1 is essential for heterochromatin formation and TE silencing, but ADCP1 itself is only a histone binding protein without any repressor domain (Zhao et al., 2019), other chromatin modeling proteins must be recruited by ADCP1 to heterochromatin. Recently, SMC4, a core subunit of condensins I and II, was identified to act in conjunction with CG methylation, CHG methylation, the chromatin remodeler DDM1 (DECREASE IN DNA METHYLATION 1), and histone modifications, including H3K9me2 and H3K27me1 (Wang et al., 2017). Considering the function of SMC4 in H3K9me2-mediated transcriptional silencing (Wang et al., 2017), it is worth knowing whether SMC4 works together with ADCP1 to mediate heterochromatic silencing. Another possibility is that ADCP1 mediates downstream silencing by driving nucleosome phase separation. It has been demonstrated that human HP1α and *Drosophila* HP1a may demix from aqueous solution to form phase-separated droplets (Larson et al., 2017; Strom et al., 2017), which rapidly induce compacted chromatin. Similarly, ADCP1 can mediate heterochromatin phase separation together with reconstituted nucleosomes bearing H3K9me3 *in vitro* (Zhao et al., 2019). Thus, ADCP1 probably has a similar ability to mediate phase separation as the functional analog of mammalian HP1.

## Role of H3K9 Methylation in *Arabidopsis* Development

The mutant with compromised KYP, SUVH5, and SUVH6 has no obvious abnormality in development; thus, H3K9 was considered to play minor roles in *Arabidopsis* development. Nevertheless, the role of H3K9me2 in *Arabidopsis* development has been identified with more careful observations and new approaches.

The main H3K9me2 methyltransferase, KYP, was proven to repress primary seed dormancy by suppressing the expression of dormancy and ABA pathway-related genes, such as *DOG1*, which is a master regulator in the control of seed dormancy (Bentsink et al., 2006), and *ABI3* and *ABI4*, which are components of ABA signaling (Koornneef et al., 2002; Zheng et al., 2012). However, evidence that H3K9me2 directly regulates the expression of these genes *via* H3K9me2 levels is not available. Until recently, SUVH5 was revealed to directly repress the expression of genes related to the ABA signaling pathway, *DOG*1, and its homologs *via* H3K9me2 in light-mediated seed germination (Gu et al., 2019). Thus, SUVH5-mediated H3K9me2 directly participates in controlling seed germination in *Arabidopsis*. After seed germination, plants enter the vegetative stage. While the role of H3K9me2 in the vegetative stage is not clear, H3K9me2 is crucial for the transition to flowering. Knockdown of *AHL16* leads to obvious late flowering, which results in increased expression of two flowering repressors, *FLOWERING LOCUS C* (*FLC*) and FWA. Consistent with the increased expression pattern, the H3K9me2 levels at the intron of *FLC* and *FWA* loci were reduced. Interestingly, the phenotype of late flowering in the *ahl16* mutant only occurs in Landsberg (Ler) accession but not in Columbia (Col) accession, indicating the ecotype-dependent regulation process (Xu et al., 2013).

During male meiosis, H3K9me2 is crucial for the distribution of meiotic recombination (Underwood et al., 2018). In plants, meiotic recombination is enriched in euchromatic regions, rather than pericentromeric heterochromatin, associated with H3K4me3 and histone variant H2A.Z but inversely correlated with DNA methylation. Suppression of meiotic recombination within the centromeric region is thought to be important for maintaining the fidelity of genome transmission during meiosis (Choi et al., 2018). Loss of DNA methylation in the *met1* mutant leads to epigenetic activation of meiotic double-strand breaks (DSBs) in proximity to centromeres (Choi et al., 2018). In addition, non-CG methylation and the H3K9me2 pathway are also responsible for suppressing pericentromeric recombination (Underwood et al., 2018). Epigenetic activation of recombination and crossovers (COs) can be induced *via* loss of H3K9me2 and non-CG methylation in the *kyp*, *suvh5*, *suvh6*, or *cmt3* mutant (Underwood et al., 2018), making it possible to induce COs near centromeres, which are otherwise very low-frequency CO regions in *Arabidopsis* and crops (Taagen et al., 2020).

In *Arabidopsis thaliana* and *Arabidopsis lyrata* seed development, H3K9me2 and CHG methylation are involved in the regulation of genomic imprinting that leads to differential expression of parent-of-origin alleles by maintaining or reinforcing the repression of maternal alleles of imprinted paternally expressed genes (PEGs) (Klosinska et al., 2016; Moreno-Romero et al., 2019). Moreover, the presence of the three repressive epigenetic marks H3K27me3, H3K9me2, and CHG methylation on the maternal alleles in endosperm can be considered a specific epigenetic signature of paternally expressed imprinted genes in the endosperm of *Arabidopsis* (Moreno-Romero et al., 2019). These marks are able to predict known PEGs at high accuracy and identify several new PEGs that were confirmed by INTACT-based endosperm transcriptomes (Moreno-Romero et al., 2019). In addition to maintaining genomic imprinting in the endosperm of diploid *Arabidopsis* seeds, H3K9me2 also functions in establishing a hybridization barrier from interploidy cross in the endosperm of triploid seeds (the triploid block) (Jiang et al., 2017). The triploid block acts as an instant reproductive barrier that prevents backcrossing of the newly formed polyploid plants with their progenitors (Schatlowski and Kohler, 2012). Multiple PEGs are enhanced in the endosperm of triploid seeds (Kradolfer et al., 2013; Wolff et al., 2015). Increased H3K9me2 levels in AT-rich TEs derived from overexpressed ADM and AHL10 contribute to enhancing the expression of PEGs, such as PEG2, which is a crucial component in establishing the triploid block. Moreover, H3K9me2 levels in AT-rich TEs are also associated with the different phenotypes of the triploid block in Col and Ler accessions.

## Conclusion and Perspective

In plants, H3K9 methylation, mainly H3K9me2, functions importantly in suppressing TEs and repetitive sequences, protecting plant genomes from TE transposition and genome instability. To enable plants to correctly deposit H3K9me2 in the genome, multiple H3K9 methyltransferases are in charge of H3K9me2 deposition in different sequence contexts *via* DNA methylation-dependent and -independent activities. Apart from playing a role in genome stability, H3K9me2 also plays roles in plant development and environmental stimuli. Recent studies have enhanced our understanding of the structure and recruitment of H3K9 methyltransferases and the downstream effector of H3K9me2, but open questions remain.

Given that H3K9me2 plays important roles in plant development and environmental stress, how the H3K9 methylation pathway is in response to developmental cues or environmental stimuli will be highly interesting to explore. In addition, our mechanistic understanding of downstream effectors of H3K9 methylation is also limited, while the H3K9me reader has been identified in *Arabidopsis*. The mechanism by which ADCP1 mediates transcriptional silencing, the existence of other H3K9me downstream effectors, the role of phase separation in chromatin condensation *in vivo*, and how H3K9me functions in response to developmental cues or environmental stimuli remains to be elucidated. Answering these questions will further broaden our understanding of H3K9 methylation-dependent transcriptional silencing.

## Author Contributions

Both authors listed have made a substantial, direct and intellectual contribution to the work, and approved it for publication.

## Conflict of Interest

The authors declare that the research was conducted in the absence of any commercial or financial relationships that could be construed as a potential conflict of interest.

## References

[B1] Arita K. Isogai S. Oda T. Unoki M. Sugita K. Sekiyama N. (2012). Recognition of modification status on a histone H3 tail by linked histone reader modules of the epigenetic regulator UHRF1. *Proc. Natl. Acad. Sci. U.S.A.* 109 12950–12955. 10.1073/pnas.1203701109 22837395 PMC3420164

[B2] Bannister A. J. Zegerman P. Partridge J. F. Miska E. A. Thomas J. O. Allshire R. C. (2001). Selective recognition of methylated lysine 9 on histone H3 by the HP1 chromo domain. *Nature* 410 120–124. 10.1038/35065138 11242054

[B3] Baumbusch L. O. Thorstensen T. Krauss V. Fischer A. Naumann K. Assalkhou R. (2001). The *Arabidopsis thaliana* genome contains at least 29 active genes encoding SET domain proteins that can be assigned to four evolutionarily conserved classes. *Nucleic Acids Res.* 29 4319–4333. 10.1093/nar/29.21.4319 11691919 PMC60187

[B4] Bentsink L. Jowett J. Hanhart C. J. Koornneef M. (2006). Cloning of *DOG1*, a quantitative trait locus controlling seed dormancy in *Arabidopsis*. *Proc. Natl. Acad. Sci. U.S.A.* 103 17042–17047. 10.1073/pnas.0607877103 17065317 PMC1636575

[B5] Bernatavichute Y. V. Zhang X. Cokus S. Pellegrini M. Jacobsen S. E. (2008). Genome-wide association of histone H3 lysine nine methylation with CHG DNA methylation in *Arabidopsis thaliana*. *PLoS One* 3:e3156. 10.1371/journal.pone.0003156 18776934 PMC2522283

[B6] Bian C. Chen Q. Yu X. (2015). The zinc finger proteins ZNF644 and WIZ regulate the G9a/GLP complex for gene repression. *eLife* 4:e05606. 10.7554/eLife.08168 25789554 PMC4365668

[B7] Cao X. Jacobsen S. E. (2002). Role of the arabidopsis DRM methyltransferases in de novo DNA methylation and gene silencing. *Curr. Biol.* 12 1138–1144. 10.1016/s0960-9822(02)00925-9 12121623

[B8] Caro E. Stroud H. Greenberg M. V. Bernatavichute Y. V. Feng S. Groth M. (2012). The SET-domain protein SUVR5 mediates H3K9me2 deposition and silencing at stimulus response genes in a DNA methylation-independent manner. *PLoS Genet.* 8:e1002995. 10.1371/journal.pgen.1002995 23071452 PMC3469426

[B9] Charron J. B. He H. Elling A. A. Deng X. W. (2009). Dynamic landscapes of four histone modifications during deetiolation in *Arabidopsis*. *Plant Cell* 21 3732–3748. 10.1105/tpc.109.066845 20008096 PMC2814509

[B10] Chen X. El Gazzar M. Yoza B. K. Mccall C. E. (2009). The NF-kappaB factor RelB and histone H3 lysine methyltransferase G9a directly interact to generate epigenetic silencing in endotoxin tolerance. *J. Biol. Chem.* 284 27857–27865. 10.1074/jbc.M109.000950 19690169 PMC2788836

[B11] Cheng J. Yang Y. Fang J. Xiao J. Zhu T. Chen F. (2013). Structural insight into coordinated recognition of trimethylated histone H3 lysine 9 (H3K9me3) by the plant homeodomain (PHD) and tandem tudor domain (TTD) of UHRF1 (ubiquitin-like, containing PHD and RING finger domains, 1) protein. *J. Biol. Chem.* 288 1329–1339. 10.1074/jbc.M112.415398 23161542 PMC3543016

[B12] Choi K. Zhao X. Tock A. J. Lambing C. Underwood C. J. Hardcastle T. J. (2018). Nucleosomes and DNA methylation shape meiotic DSB frequency in *Arabidopsis thaliana* transposons and gene regulatory regions. *Genome Res.* 28 532–546. 10.1101/gr.225599.117 29530928 PMC5880243

[B13] Ding Y. Wang X. Su L. Zhai J. Cao S. Zhang D. (2007). SDG714, a histone H3K9 methyltransferase, is involved in Tos17 DNA methylation and transposition in rice. *Plant Cell* 19 9–22. 10.1105/tpc.106.048124 17259261 PMC1820975

[B14] Dodge J. E. Kang Y. K. Beppu H. Lei H. Li E. (2004). Histone H3-K9 methyltransferase ESET is essential for early development. *Mol. Cell. Biol.* 24 2478–2486. 10.1128/mcb.24.6.2478-2486.2004 14993285 PMC355869

[B15] Du J. Zhong X. Bernatavichute Y. V. Stroud H. Feng S. Caro E. (2012). Dual binding of chromomethylase domains to H3K9me2-containing nucleosomes directs DNA methylation in plants. *Cell* 151 167–180. 10.1016/j.cell.2012.07.034 23021223 PMC3471781

[B16] Du J. M. Johnson L. M. Groth M. Feng S. H. Hale C. J. Li S. S. (2014). Mechanism of DNA methylation-directed histone methylation by KRYPTONITE. *Mol. Cell* 55 495–504. 10.1016/j.molcel.2014.06.009 25018018 PMC4127122

[B17] Du J. M. Johnson L. M. Jacobsen S. E. Patel D. J. (2015). DNA methylation pathways and their crosstalk with histone methylation. *Nat. Rev. Mol. Cell Biol.* 16 519–532. 10.1038/nrm4043 26296162 PMC4672940

[B18] Ebbs M. L. Bender J. (2006). Locus-specific control of DNA methylation by the *Arabidopsis* SUVH5 histone methyltransferase. *Plant Cell* 18 1166–1176. 10.1105/tpc.106.041400 16582009 PMC1456864

[B19] Espada J. Ballestar E. Fraga M. F. Garea A. V. Juarranz A. Stockert J. C. (2004). Human DNA methyltransferase 1 is required for maintenance of the histone H3 modification pattern. *J. Biol. Chem.* 279 37175–37184. 10.1074/jbc.M404842200 15220328

[B20] Exner V. Aichinger E. Shu H. Wildhaber T. Alfarano P. Caflisch A. (2009). The Chromodomain of LIKE HETEROCHROMATIN PROTEIN 1 is Essential for H3K27me3 binding and function during arabidopsis development. *PLoS One* 4:e5335. 10.1371/journal.pone.0005335 19399177 PMC2670505

[B21] Finnegan E. J. Peacock W. J. Dennis E. S. (1996). Reduced DNA methylation in *Arabidopsis thaliana* results in abnormal plant development. *Proc. Natl. Acad. Sci. U.S.A.* 93 8449–8454. 10.1073/pnas.93.16.8449 8710891 PMC38691

[B22] Fog C. K. Galli G. G. Lund A. H. (2012). PRDM proteins: important players in differentiation and disease. *Bioessays* 34 50–60. 10.1002/bies.201100107 22028065

[B23] Gehring M. Missirian V. Henikoff S. (2011). Genomic analysis of parent-of-origin allelic expression in *Arabidopsis thaliana* seeds. *PLoS One* 6:e23687. 10.1371/journal.pone.0023687 21858209 PMC3157454

[B24] Gu D. C. Ji R. J. He C. M. Peng T. Zhang M. Y. Duan J. (2019). *Arabidopsis* histone methyltransferase SUVH5 is a positive regulator of light-mediated seed germination. *Front. Plant Sci.* 10:841. 10.3389/fpls.2019.00841 31316539 PMC6610342

[B25] Gu X. Jiang D. Yang W. Jacob Y. Michaels S. D. He Y. (2011). *Arabidopsis* homologs of retinoblastoma-associated protein 46/48 associate with a histone deacetylase to act redundantly in chromatin silencing. *PLoS Genet.* 7:e1002366. 10.1371/journal.pgen.1002366 22102827 PMC3213158

[B26] Han Y. F. Dou K. Ma Z. Y. Zhang S. W. Huang H. W. Li L. (2014). SUVR2 is involved in transcriptional gene silencing by associating with SNF2-related chromatin-remodeling proteins in *Arabidopsis*. *Cell Res.* 24 1445–1465. 10.1038/cr.2014.156 25420628 PMC4260354

[B27] Harris C. J. Scheibe M. Wongpalee S. P. Liu W. L. Cornett E. M. Vaughan R. M. (2018). A DNA methylation reader complex that enhances gene transcription. *Science* 362 1182–1186. 10.1126/science.aar7854 30523112 PMC6353633

[B28] Honda S. Bicocca V. T. Gessaman J. D. Rountree M. R. Yokoyama A. Yu E. Y. (2016). Dual chromatin recognition by the histone deacetylase complex HCHC is required for proper DNA methylation in Neurospora crassa. *Proc. Natl. Acad. Sci. U.S.A.* 113 E6135–E6144. 10.1073/pnas.1621475114 27681634 PMC5068333

[B29] Huo X. R. Ji L. Z. Zhang Y. W. Lv P. Cao X. Wang Q. F. (2020). The nuclear matrix protein SAFB cooperates with major satellite RNAs to stabilize heterochromatin architecture partially through phase separation. *Mol. Cell* 77 368–383.e7. 10.1016/j.molcel.2019.10.001 31677973

[B30] Ikegami K. Iwatani M. Suzuki M. Tachibana M. Shinkai Y. Tanaka S. (2007). Genome-wide and locus-specific DNA hypomethylation in G9a deficient mouse embryonic stem cells. *Genes Cells* 12 1–11. 10.1111/j.1365-2443.2006.01029.x 17212651

[B31] Jackson J. P. Johnson L. Jasencakova Z. Zhang X. Perezburgos L. Singh P. B. (2004). Dimethylation of histone H3 lysine 9 is a critical mark for DNA methylation and gene silencing in *Arabidopsis thaliana*. *Chromosoma* 112 308–315. 10.1007/s00412-004-0275-7 15014946

[B32] Jackson J. P. Lindroth A. M. Cao X. Jacobsen S. E. (2002). Control of CpNpG DNA methylation by the KRYPTONITE histone H3 methyltransferase. *Nature* 416 556–560. 10.1038/nature731 11898023

[B33] Jacobs S. A. Taverna S. D. Zhang Y. Briggs S. D. Li J. Eissenberg J. C. (2001). Specificity of the HP1 chromo domain for the methylated N-terminus of histone H3. *EMBO J.* 20 5232–5241. 10.1093/emboj/20.18.5232 11566886 PMC125272

[B34] Jenuwein T. Allis C. D. (2001). Translating the histone code. *Science* 293 1074–1080. 11498575 10.1126/science.1063127

[B35] Jenuwein T. Laible G. Dorn R. Reuter G. (1998). SET domain proteins modulate chromatin domains in eu- and heterochromatin. *Cell. Mol. Life Sci.* 54 80–93. 10.1007/s000180050127 9487389 PMC11147257

[B36] Jiang H. Moreno-Romero J. Santos-Gonzalez J. De Jaeger G. Gevaert K. Van De Slijke E. (2017). Ectopic application of the repressive histone modification H3K9me2 establishes post-zygotic reproductive isolation in *Arabidopsis thaliana*. *Genes Dev*. 31 1272–1287. 10.1101/gad.299347.117 28743695 PMC5558928

[B37] Jing Y. Sun H. Yuan W. Wang Y. Li Q. Liu Y. (2016). SUVH2 and SUVH9 couple two essential steps for transcriptional gene silencing in *Arabidopsis*. *Mol. Plant* 9 1156–1167. 10.1016/j.molp.2016.05.006 27216319

[B38] Johnson L. M. Bostick M. Zhang X. Kraft E. Henderson I. Callis J. (2007). The SRA methyl-cytosine-binding domain links DNA and histone methylation. *Curr. Biol.* 17 379–384. 10.1016/j.cub.2007.01.009 17239600 PMC1850948

[B39] Johnson L. M. Du J. Hale C. J. Bischof S. Feng S. Chodavarapu R. K. (2014). SRA- and SET-domain-containing proteins link RNA polymerase V occupancy to DNA methylation. *Nature* 507 124–128. 10.1038/nature12931 24463519 PMC3963826

[B40] Johnson L. M. Law J. A. Khattar A. Henderson I. R. Jacobsen S. E. (2008). SRA-domain proteins required for DRM2-mediated de novo DNA methylation. *PLoS Genet.* 4:e1000280. 10.1371/journal.pgen.1000280 19043555 PMC2582956

[B41] Kim K. C. Huang S. (2003). Histone methyltransferases in tumor suppression. *Cancer Biol. Ther.* 2 491–499. 14614313 10.4161/cbt.2.5.629

[B42] Klosinska M. Picard C. L. Gehring M. (2016). Conserved imprinting associated with unique epigenetic signatures in the Arabidopsis genus. *Nat. Plants* 2:16145. 10.1038/nplants.2016.145 27643534 PMC5367468

[B43] Koornneef M. Bentsink L. Hilhorst H. (2002). Seed dormancy and germination. *Curr. Opin. Plant Biol.* 5 33–36. 11788305 10.1016/s1369-5266(01)00219-9

[B44] Kradolfer D. Wolff P. Jiang H. Siretskiy A. Kohler C. (2013). An imprinted gene underlies postzygotic reproductive isolation in *Arabidopsis thaliana*. *Dev. Cell* 26 525–535. 10.1016/j.devcel.2013.08.006 24012484

[B45] Kramer J. M. (2016). Regulation of cell differentiation and function by the euchromatin histone methyltranserfases G9a and GLP. *Biochem. Cell Biol.* 94 26–32. 10.1139/bcb-2015-0017 26198080

[B46] Larson A. G. Elnatan D. Keenen M. M. Trnka M. J. Johnston J. B. Burlingame A. L. (2017). Liquid droplet formation by HP1alpha suggests a role for phase separation in heterochromatin. *Nature* 547 236–240. 10.1038/nature22822 28636604 PMC5606208

[B47] Law J. A. Du J. M. Hale C. J. Feng S. H. Krajewski K. Palanca A. M. S. (2013). Polymerase IV occupancy at RNA-directed DNA methylation sites requires SHH1. *Nature* 498 385–389. 10.1038/nature12178 23636332 PMC4119789

[B48] Lei L. Zhou S. L. Ma H. Zhang L. S. (2012). Expansion and diversification of the SET domain gene family following whole-genome duplications in *Populus trichocarpa*. *BMC Evol. Biol.* 12:51. 10.1186/1471-2148-12-51 22497662 PMC3402991

[B49] Leung D. C. Dong K. B. Maksakova I. A. Goyal P. Appanah R. Lee S. (2011). Lysine methyltransferase G9a is required for de novo DNA methylation and the establishment, but not the maintenance, of proviral silencing. *Proc. Natl. Acad. Sci. U.S.A.* 108 5718–5723. 10.1073/pnas.1014660108 21427230 PMC3078371

[B50] Li G. Ji T. Chen J. Fu Y. Hou L. Feng Y. (2017). CRL4(DCAF8) ubiquitin ligase targets histone H3K79 and promotes H3K9 methylation in the liver. *Cell Rep.* 18 1499–1511. 10.1016/j.celrep.2017.01.039 28178526

[B51] Li X. Harris C. J. Zhong Z. Chen W. Liu R. Jia B. (2018). Mechanistic insights into plant SUVH family H3K9 methyltransferases and their binding to context-biased non-CG DNA methylation. *Proc. Natl. Acad. Sci. U.S.A.* 115 E8793–E8802. 10.1073/pnas.1809841115 30150382 PMC6140468

[B52] Li Y. H. Kirschmann D. A. Wallrath L. L. (2002). Does heterochromatin protein 1 always follow code? *Proc. Natl. Acad. Sci. U.S.A.* 99 16462–16469. 10.1073/pnas.162371699 12151603 PMC139909

[B53] Lindroth A. M. Cao X. Jackson J. P. Zilberman D. Mccallum C. M. Henikoff S. (2001). Requirement of CHROMOMETHYLASE3 for maintenance of CpXpG methylation. *Science* 292 2077–2080. 10.1126/science.1059745 11349138

[B54] Liu C. Yu Y. Liu F. Wei X. Wrobel J. A. Gunawardena H. P. (2014). A chromatin activity-based chemoproteomic approach reveals a transcriptional repressome for gene-specific silencing. *Nat. Commun.* 5:5733. 10.1038/ncomms6733 25502336 PMC4360912

[B55] Liu X. Yang S. Zhao M. Luo M. Yu C. W. Chen C. Y. (2014). Transcriptional repression by histone deacetylases in plants. *Mol. Plant* 7 764–772. 10.1093/mp/ssu033 24658416

[B56] Liu Z. W. Shao C. R. Zhang C. J. Zhou J. X. Zhang S. W. Li L. (2014). The SET domain proteins SUVH2 and SUVH9 are required for Pol V Occupancy at RNA-Directed DNA methylation loci. *PLoS Genet.* 10:e1003948. 10.1371/journal.pgen.1003948 24465213 PMC3898904

[B57] Lu F. L. Cui X. Zhang S. B. Jenuwein T. Cao X. F. (2011). Arabidopsis REF6 is a histone H3 lysine 27 demethylase. *Nat. Genet.* 43 715–719. 10.1038/ng.854 21642989

[B58] Moreno-Romero J. Del Toro-De Leon G. Yadav V. K. Santos-Gonzalez J. Kohler C. (2019). Epigenetic signatures associated with imprinted paternally expressed genes in the Arabidopsis endosperm. *Genome Biol.* 20:41. 10.1186/s13059-019-1652-0 30791924 PMC6385439

[B59] Nakayama J. Rice J. C. Strahl B. D. Allis C. D. Grewal S. I. (2001). Role of histone H3 lysine 9 methylation in epigenetic control of heterochromatin assembly. *Science* 292 110–113. 10.1126/science.1060118 11283354

[B60] Naumann K. Fischer A. Hofmann I. Krauss V. Phalke S. Irmler K. (2005). Pivotal role of AtSUVH2 in heterochromatic histone methylation and gene silencing in *Arabidopsis*. *EMBO J.* 24 1418–1429. 10.1038/sj.emboj.7600604 15775980 PMC1142535

[B61] Peters A. H. Kubicek S. Mechtler K. O’sullivan R. J. Derijck A. A. Perez-Burgos L. (2003). Partitioning and plasticity of repressive histone methylation states in mammalian chromatin. *Mol. Cell* 12 1577–1589. 10.1016/s1097-2765(03)00477-5 14690609

[B62] Rice J. C. Briggs S. D. Ueberheide B. Barber C. M. Shabanowitz J. Hunt D. F. (2003). Histone methyltransferases direct different degrees of methylation to define distinct chromatin domains. *Mol. Cell* 12 1591–1598. 10.1016/s1097-2765(03)00479-9 14690610

[B63] Roudier F. Teixeira F. K. Colot V. (2009). Chromatin indexing in Arabidopsis: an epigenomic tale of tails and more. *Trends Genet.* 25 511–517. 10.1016/j.tig.2009.09.013 19850370

[B64] Saksouk N. Simboeck E. Dejardin J. (2015). Constitutive heterochromatin formation and transcription in mammals. *Epigenet. Chromatin* 8:3. 10.1186/1756-8935-8-3 25788984 PMC4363358

[B65] Schatlowski N. Kohler C. (2012). Tearing down barriers: understanding the molecular mechanisms of interploidy hybridizations. *J. Exp. Bot.* 63 6059–6067. 10.1093/jxb/ers288 23105129

[B66] Scheer S. Zaph C. (2017). The lysine methyltransferase G9a in immune cell differentiation and function. *Front. Immunol.* 8:429. 10.3389/fimmu.2017.00429 28443098 PMC5387087

[B67] Shin J. H. Wang H. L. Lee J. Dinwiddie B. L. Belostotsky D. A. Chekanova J. A. (2013). The role of the Arabidopsis Exosome in siRNA-independent silencing of heterochromatic loci. *PLoS Genet.* 9:e1003411. 10.1371/journal.pgen.1003411 23555312 PMC3610620

[B68] Shinkai Y. Tachibana M. (2011). H3K9 methyltransferase G9a and the related molecule GLP. *Genes Dev.* 25 781–788. 10.1101/gad.2027411 21498567 PMC3078703

[B69] Sims R. J. III Nishioka K. Reinberg D. (2003). Histone lysine methylation: a signature for chromatin function. *Trends Genet.* 19 629–639. 10.1016/j.tig.2003.09.007 14585615

[B70] Strom A. R. Emelyanov A. V. Mir M. Fyodorov D. V. Darzacq X. Karpen G. H. (2017). Phase separation drives heterochromatin domain formation. *Nature* 547 241–245. 10.1038/nature22989 28636597 PMC6022742

[B71] Stroud H. Do T. Du J. Zhong X. Feng S. Johnson L. (2014). Non-CG methylation patterns shape the epigenetic landscape in Arabidopsis. *Nat. Struct. Mol. Biol.* 21 64–72. 10.1038/nsmb.2735 24336224 PMC4103798

[B72] Taagen E. Bogdanove A. J. Sorrells M. E. (2020). Counting on crossovers: controlled recombination for plant breeding. *Trends Plant Sci.* 10.1016/j.tplants.2019.12.017 [Epub ahead of print] 31959421

[B73] Tamaru H. Selker E. U. (2003). Synthesis of signals for de novo DNA methylation in *Neurospora crassa*. *Mol. Cell Biol.* 23 2379–2394. 10.1128/mcb.23.7.2379-2394.2003 12640122 PMC150737

[B74] Tetko I. V. Haberer G. Rudd S. Meyers B. Mewes H. W. Mayer K. F. (2006). Spatiotemporal expression control correlates with intragenic scaffold matrix attachment regions (S/MARs) in *Arabidopsis thaliana*. *PLoS Comput. Biol.* 2:e21. 10.1371/journal.pcbi.0020021 16604187 PMC1420657

[B75] Thorstensen T. Fischer A. Sandvik S. V. Johnsen S. S. Grini P. E. Reuter G. (2006). The Arabidopsis SUVR4 protein is a nucleolar histone methyltransferase with preference for monomethylated H3K9. *Nucleic Acids Res.* 34 5461–5470. 10.1093/nar/gkl687 17020925 PMC1636477

[B76] Trojer P. Reinberg D. (2007). Facultative heterochromatin: Is there a distinctive molecular signature? *Mol. Cell* 28 1–13. 10.1016/j.molcel.2007.09.011 17936700

[B77] Turck F. Roudier F. Farrona S. Martin-Magniette M. L. Guillaume E. Buisine N. (2007). Arabidopsis TFL2/LHP1 specifically associates with genes marked by trimethylation of histone H3 lysine 27. *PLoS Genet.* 3:e86. 10.1371/journal.pgen.0030086 17542647 PMC1885283

[B78] Underwood C. J. Choi K. Lambing C. Zhao X. Serra H. Borges F. (2018). Epigenetic activation of meiotic recombination near *Arabidopsis thaliana* centromeres via loss of H3K9me2 and non-CG DNA methylation. *Genome Res.* 28 519–531. 10.1101/gr.227116.117 29530927 PMC5880242

[B79] Vaute O. Nicolas E. Vandel L. Trouche D. (2002). Functional and physical interaction between the histone methyl transferase Suv39H1 and histone deacetylases. *Nucleic Acids Res.* 30 475–481. 10.1093/nar/30.2.475 11788710 PMC99834

[B80] Veiseth S. V. Rahman M. A. Yap K. L. Fischer A. Egge-Jacobsen W. Reuter G. (2011). The SUVR4 histone lysine methyltransferase binds ubiquitin and converts H3K9me1 to H3K9me3 on transposon chromatin in Arabidopsis. *PLoS Genet.* 7:e1001325. 10.1371/journal.pgen.1001325 21423664 PMC3053343

[B81] Wang J. Blevins T. Podicheti R. Haag J. R. Tan E. H. Wang F. (2017). Mutation of Arabidopsis SMC4 identifies condensin as a corepressor of pericentromeric transposons and conditionally expressed genes. *Genes Dev.* 31 1601–1614. 10.1101/gad.301499.117 28882854 PMC5630024

[B82] Wierzbicki A. T. Haag J. R. Pikaard C. S. (2008). Noncoding transcription by RNA Polymerase Pol IVb/Pol V mediates transcriptional silencing of overlapping and adjacent genes. *Cell* 135 635–648. 10.1016/j.cell.2008.09.035 19013275 PMC2602798

[B83] Wolff P. Jiang H. Wang G. Santos-Gonzalez J. Kohler C. (2015). Paternally expressed imprinted genes establish postzygotic hybridization barriers in *Arabidopsis thaliana*. *eLife* 4:e10074. 10.7554/eLife.10074 26344545 PMC4589659

[B84] Wolff P. Weinhofer I. Seguin J. Roszak P. Beisel C. Donoghue M. T. (2011). High-resolution analysis of parent-of-origin allelic expression in the Arabidopsis Endosperm. *PLoS Genet.* 7:e1002126. 10.1371/journal.pgen.1002126 21698132 PMC3116908

[B85] Wu H. Min J. Lunin V. V. Antoshenko T. Dombrovski L. Zeng H. (2010). Structural biology of human H3K9 methyltransferases. *PLoS One* 5:e8570. 10.1371/journal.pone.0008570 20084102 PMC2797608

[B86] Xiao C. Chen F. Yu X. Lin C. Fu Y. F. (2009). Over-expression of an AT-hook gene, AHL22, delays flowering and inhibits the elongation of the hypocotyl in *Arabidopsis thaliana*. *Plant Mol. Biol.* 71 39–50. 10.1007/s11103-009-9507-9 19517252

[B87] Xiao X. Zhang J. Li T. Fu X. Satheesh V. Niu Q. (2019). A group of SUVH methyl-DNA binding proteins regulate expression of the DNA demethylase ROS1 in *Arabidopsis*. *J. Integr. Plant Biol.* 61 110–119. 10.1111/jipb.12768 30589237

[B88] Xu Y. F. Wang Y. Z. Stroud H. Gu X. F. Sun B. Gan E. S. (2013). A matrix protein silences transposons and repeats through interaction with retinoblastoma-associated proteins. *Curr. Biol.* 23 345–350. 10.1016/j.cub.2013.01.030 23394836

[B89] Yoshida K. Maekawa T. Zhu Y. Renard-Guillet C. Chatton B. Inoue K. (2015). The transcription factor ATF7 mediates lipopolysaccharide-induced epigenetic changes in macrophages involved in innate immunological memory. *Nat. Immunol.* 16 1034–1043. 10.1038/ni.3257 26322480

[B90] Yu C. W. Tai R. Wang S. C. Yang P. Luo M. Yang S. (2017). HISTONE DEACETYLASE6 acts in concert with histone methyltransferases SUVH4, SUVH5, and SUVH6 to regulate transposon silencing. *Plant Cell* 29 1970–1983. 10.1105/tpc.16.00570 28778955 PMC5590490

[B91] Yun J. Kim Y. S. Jung J. H. Seo P. J. Park C. M. (2012). The AT-hook motif-containing protein AHL22 regulates flowering initiation by modifying *FLOWERING LOCUS T* chromatin in *Arabidopsis*. *J. Biol. Chem.* 287 15307–15316. 10.1074/jbc.M111.318477 22442143 PMC3346147

[B92] Zemach A. Kim M. Y. Hsieh P. H. Coleman-Derr D. Eshed-Williams L. Thao K. (2013). The Arabidopsis nucleosome remodeler DDM1 allows DNA methyltransferases to access H1-containing heterochromatin. *Cell* 153 193–205. 10.1016/j.cell.2013.02.033 23540698 PMC4035305

[B93] Zeng W. Ball A. R. Jr. Yokomori K. (2010). HP1: heterochromatin binding proteins working the genome. *Epigenetics* 5 287–292. 10.4161/epi.5.4.11683 20421743 PMC3103764

[B94] Zhang C. Du X. Tang K. Yang Z. Pan L. Zhu P. (2018). Arabidopsis AGDP1 links H3K9me2 to DNA methylation in heterochromatin. *Nat. Commun.* 9:4547. 10.1038/s41467-018-06965-w 30382101 PMC6208443

[B95] Zhang L. S. Ma H. (2012). Complex evolutionary history and diverse domain organization of SET proteins suggest divergent regulatory interactions. *New Phytol.* 195 248–263. 10.1111/j.1469-8137.2012.04143.x 22510098

[B96] Zhang Y. Harris C. J. Liu Q. Liu W. Ausin I. Long Y. (2018). Large-scale comparative epigenomics reveals hierarchical regulation of non-CG methylation in *Arabidopsis*. *Proc. Natl. Acad. Sci. U.S.A.* 115 E1069–E1074. 10.1073/pnas.1716300115 29339507 PMC5798360

[B97] Zhao J. F. Favero D. S. Qiu J. W. Roalson E. H. Neff M. M. (2014). Insights into the evolution and diversification of the AT-hook Motif Nuclear Localized gene family in land plants. *BMC Plant Biol.* 14:266. 10.1186/s12870-014-0266-7 25311531 PMC4209074

[B98] Zhao S. Cheng L. Gao Y. Zhang B. Zheng X. Wang L. (2019). Plant HP1 protein ADCP1 links multivalent H3K9 methylation readout to heterochromatin formation. *Cell Res.* 29 54–66. 10.1038/s41422-018-0104-9 30425322 PMC6318295

[B99] Zheng B. Wang Z. Li S. Yu B. Liu J. Y. Chen X. (2009). Intergenic transcription by RNA polymerase II coordinates Pol IV and Pol V in siRNA-directed transcriptional gene silencing in *Arabidopsis*. *Genes Dev.* 23 2850–2860. 10.1101/gad.1868009 19948763 PMC2800093

[B100] Zheng J. Chen F. Wang Z. Cao H. Li X. Deng X. (2012). A novel role for histone methyltransferase KYP/SUVH4 in the control of Arabidopsis primary seed dormancy. *New Phytol.* 193 605–616. 10.1111/j.1469-8137.2011.03969.x 22122546

[B101] Zhou J. Wang X. He K. Charron J. B. Elling A. A. Deng X. W. (2010). Genome-wide profiling of histone H3 lysine 9 acetylation and dimethylation in Arabidopsis reveals correlation between multiple histone marks and gene expression. *Plant Mol. Biol.* 72 585–595. 10.1007/s11103-009-9594-7 20054610

